# Midazolam exhibits antitumour and enhances the efficiency of Anti-PD-1 immunotherapy in hepatocellular carcinoma

**DOI:** 10.1186/s12935-022-02735-3

**Published:** 2022-10-12

**Authors:** Junwei Kang, Zhiying Zheng, Xian Li, Tian Huang, Dawei Rong, Xinyang Liu, Miaomiao Qin, Yuliang Wang, Xiangyi Kong, Jinhua Song, Chengyu Lv, Xiongxiong Pan

**Affiliations:** 1 Department of Anesthesiology and Perioperative Medicine, The First Affiliated Hospital of Nanjing Medical University, Nanjing, China; 2 Department of General Surgery, Nanjing First Hospital, Nanjing Medical University, Nanjing, China; 3 Hepatobiliary Center, Key Laboratory of Liver Transplantation, NHC Key Laboratory of Living Donor Liver Transplantation, The First Affiliated Hospital of Nanjing Medical University, Chinese Academy of Medical Sciences, Nanjing, China; 4 Basic Medical School, Nanjing Medical University, Nanjing, China

**Keywords:** MDZ, Hepatocellular carcinoma, Immune escape, PD-L1, PD-1

## Abstract

**Background:**

Midazolam (MDZ) is an anaesthetic that is widely used for anxiolysis and sedation. More recently, MDZ has also been described to be related to the outcome of various types of carcinomas. However, how MDZ influences the progression of hepatocellular carcinoma (HCC) and its effects on the biological function and tumour immune microenvironment of this type of tumour remain unknown.

**Methods:**

The effects of MDZ on the proliferation, invasion, and migration of HCC cell lines were examined in vitro using the Cell Counting Kit 8 (CCK8), 5-ethynyl-2ʹ-deoxyuridine (EdU), Transwell, and wound healing assays. Additionally, western blotting was employed to confirm that PD-L1 was expressed. Chromatin immunoprecipitation-seq (ChIP-seq) analysis was used to pinpoint the transcriptional regulation regions of NF-κB and programmed death-ligand 1 (PD-L1). A C57BL/6 mouse model was used to produce subcutaneous HCC tumors in order to evaluate the in vivo performance of MDZ. Mass spectrometry was also used to assess changes in the tumour immunological microenvironment following MDZ injection.

**Results:**

The HCC-LM3 and Hep-3B cell lines’ proliferation, invasion, and migration were controlled by MDZ, according to the results of the CCK8, EdU, Transwell, and wound healing assays. PD-L1 expression was shown by ChIP-seq analysis to be boosted by NF-κB, and by Western blotting analysis, it was shown that MDZ downregulated the expression of NF-κB. Additionally, in vivo tests revealed that intraperitoneal MDZ injections reduced HCC tumor development and enhanced the effectiveness of anti-PD-1 therapy. The CD45^+^ immune cell proportions were higher in the MDZ group than in the PBS group, according to the mass spectrometry results. Injection of MDZ resulted in a decrease in the proportions of CD4^+^ T cells, CD8^+^ T cells, natural killer (NK) cells, monocytes, Tregs, and M2 macrophages and a rise in the proportion of dendritic cells. Additionally, the concentrations of the cytokines IFN-g and TNF-a were noticeably raised whereas the concentrations of the CD8^+^ T-cell fatigue markers ICOS, TIGIT, and TIM3 were noticeably lowered.

**Conclusion:**

According to this study, MDZ inhibited the progression of HCC by inhibiting the NF-κB pathway and reducing the exhaustion of CD8^+^ T cells. In clinical practice, MDZ combined with anti-PD-1 therapy might contribute to synergistically improving the antitumor efficacy of HCC treatment.

**Supplementary Information:**

The online version contains supplementary material available at 10.1186/s12935-022-02735-3.

## Introduction

Liver cancer (LC) is a common and lethal malignancy, but treatment approaches and the clinical management of LC face substantial challenges [1]. Liver cancer is diagnosed in more than 800,000 people worldwide and accounts for over 700,000 deaths annually [2]. Hepatocellular carcinoma (HCC), which accounts for 90% of all occurrences of LC [3], is the most common kind of preinvasive liver cancer. Clinical HCC treatment has made notable strides in recent years [4–6]. In the clinic, surgery paired with chemoradiation is an essential part of treating HCC; nonetheless, some patients’ 5-year survival rates are still low [7]. In addition, due to the atypical clinical symptoms of early HCC and the low prevalence rate of HCC early diagnosis [8], most patients miss their best chance for undergoing surgery. Therefore, exploration of potential therapeutic targets and their specific mechanisms in HCC is urgently needed.

More recently, effective tumour immunotherapy, which targets immune checkpoints, has attracted great attention. Immune checkpoints include coinhibitory and costimulatory molecules that are expressed by effector lymphocytes and prevent the development of excessive immune responses. The production of the corresponding ligands by tumor and stromal cells is a biological mechanism used by liver tumors and other malignancies to evade antitumor immune responses [9]. PD-1, T cell immunoglobulin and mucin domain containing-3 (TIM-3), cytotoxic T lymphocyte-associated antigen-4 (CTLA-4), lymphocyte-activation gene-3 (LAG-3) and others are examples of co-receptors [10]. Most Treg cells express CTLA-4, which acts as an effector molecule to prevent the proper activation of effector T cells [11]. In contrast to its ligand, PD-L1, which is expressed by a variety of stromal and tumor cells as well as myeloid cells, including DCs, PD-1 is expressed by activated T cells, natural killer (NK) cells, Treg cells, MDSCs, monocytes, and dendritic cells (DCs). Effector T cells become exhausted or become dysfunctional as a result of PD-1, which prevents effector functions. Immune checkpoint inhibitors (ICIs) work to stop the interaction between checkpoint proteins and their ligands, thereby preventing T cells from becoming inactive. The discovery that an effective immune response can partially or completely eradicate tumor cells across a variety of malignancies has transformed cancer therapy [12].

Midazolam (MDZ), which is a derivative of benzodiazepine, is extensively used for procedural sedation and the induction of general anaesthesia in clinical practice. Animal experiments have demonstrated that MDZ can inhibit the ability of animals to exercise and induce muscle relaxation [13]. In addition, many original studies have recently shown that MDZ demonstrates an extraordinary ability to promote apoptosis in several human cancer cells and hinder tumour growth in xenotransplantation mouse models [14]. However, studies on the relationship between MDZ and the tumour immune microenvironment have not been carried out. In our study, we explored the effect exerted by MDZ on HCC progression and revealed that MDZ reduces the expression of PD-L1 in HCC cell lines by suppressing the NF-κB pathway. The combination of MDZ and the PD-1 mAb is beneficial for increasing antitumor activity, which may provide novel clinical immunotherapeutic approaches for treating HCC and provide new evidence of the relationship between perioperative anaesthesia and HCC tumour progression.

## Materials and methods

### Cell culture and drug treatments

The Cell Bank of Type Culture Collection provided the human HCC-LM3 and Hep-3B HCC cell lines as well as the mouse Hepa1-6 HCC cell line (Chinese Academy of Sciences, China). HCC-LM3 and Hep-3B cells were grown in DMEM (Gibco, USA) supplemented with 10% FBS and 1% penicillin/streptomycin, whereas Hepa1-6 cells were grown in RIPA 1640 medium (Gibco, USA) with 10% FBS and 1% penicillin/streptomycin. All of the cell lines were kept in a single incubator with a constant temperature of 37 °C and 5% CO_2_. For the pertinent investigations, the HCC-LM3 and Hep-3B HCC cell lines were treated with MDZ (Nhwa Pharmaceutical, China) diluted with normal saline (0.9%) when they had reached 80–90% confluence.

### Cell proliferation assay

For the CCK8 proliferation experiments, HCC-LM3 and Hep-3B cells were meticulously seeded in 96 wells at a density of 2000 cells per well. After the cells were exposed to MDZ (0, 50, 75, 100, 150, 200 μM) for 0 h, 24 h, 48 h, and 72 h, they were then treated with 10 μl of CCK8 solution (RiboBio, China). Following that, a microplate reader was used to measure the cells’ absorbance at 450 nm in accordance with the manufacturer's instructions (Synergy, USA).

Using a Cell-Light EdU DNA Cell Proliferation Kit, the EdU assay was carried out to assess cell proliferation (RiboBio, China). In a 24-well plate, 10 × 10^4^ HCC cells were seeded in each well. After 80–90% of the cells had formed monolayers, we added MDZ (75 and 150 μM) and incubated the mixture for 24 h. The two cell lines were then fixed with 4% paraformaldehyde following a 24-h incubation with a 10 mmol/l EdU solution. Following that, we gave the cells the prescribed amounts of DAPI and Apollo Dye Solution. Using an Olympus FSX100 microscope (Olympus, Japan), photographs of the EdU-incorporated cells were taken, and the quantity of EdU-positive cells was counted.

### Transwell invasion assay

In the upper chamber of Transwell chambers (Corning, USA) containing matrix mixes (BD Biosciences, USA) and 300 μl serum-free media, HCC-LM3 and Hep-3B cells (3 × 10^4^ cells) were plated and pretreated with MDZ (75 and 150 μM) for 24 h. 600 μl of 10% FBS-supplemented media, which functioned as the HCC cells’ chemoattractant, were placed in the bottom compartment. The non-invading cells were eliminated after the 24-h incubation period, and the invaded cells were then fixed with 4% paraformaldehyde for 15 min before being stained for 20 min using Crystal Violet Staining Solution (Beyotime, China). We next used a fluorescence microscope to take pictures of the labeled cells and to count them.

### Wound healing assay

In 6-well plates, HCC-LM3 and Hep-3B cells were plated. The monolayer was then scratched using a common 20-μl pipette tip to create an artificial homogenous wound after the cells had grown into 90–100% confluent cell monolayers. The plate was then mixed with serum-free medium supplemented with 75 and 150 μM MDZ, and the thermomixer was used to incubate it at 37 °C after the free-floating cells and debris were removed using phosphate-buffered saline (PBS, Gibco, USA). After 0 h, 24 h, and 48 h, images of the wound widths were taken using an inverted microscope for additional analysis.

### Western blotting

Using a Mammalian Total Protein Extraction Kit and a protease inhibitor cocktail, the cells' total proteins were extracted (Transgen, Beijing, China). In a nutshell, 10% SDS–polyacrylamide gel electrophoresis was used to separate the proteins. Following that, the proteins were transferred to PVDF membranes. Specific primary antibodies (Abcam, UK) were incubated with the PVDF membranes for an extended period of time at 4 °C in order to identify PD-L1, P65, p-P65, and GAPDH. The membranes were then exposed to peroxidase-conjugated secondary antibodies for two hours at room temperature. Finally, an enhanced chemiluminescence assay was used to see the Western blotting bands (ECL; Thermo Fisher, USA).

### Chromatin immunoprecipitation-seq (ChIP-seq) analysis and site prediction

The JASPAR database (http://jaspar.genereg.net) was used to predict the binding sites for P65 in the promoter of the PD-L1 gene. The human P65-PD-L1 binding sites' CHIP data were obtained from GSE2360959.

### Tumour mouse models and treatment

Twenty male C57BL/6 mice, aged 5 to 6 weeks, were acquired from the Laboratory Animal Center of Nanjing Medical University and fed under specified pathogen-free (SPF) conditions. 2 × 10^6^ murine Hepa1-6 cells were subcutaneously implanted into the right axillary areas of the mice to create the subcutaneous tumor mouse model. Subsequently, 20 subcutaneous tumour-bearing mice were randomly divided to four groups: the PBS, MDZ, α-PD-1, and α-PD-1 + MDZ groups. MDZ (1 mg kg^−1^, every other day) was intraperitoneally injected into the mice in the MDZ and α-PD-1 + MDZ groups when the tumours reached approximately 50–100 mm^3^ in volume, while the PBS and α-PD-1 groups were injected with an equal dose of PBS. After 7 days, 200 µg anti-mouse-PD-1 (α-PD-1; BE0273; Bio X Cell, USA) was injected intraperitoneally into the mice in the α-PD-1 and α-PD-1 + MDZ groups twice a week. On Day 21, the tumour-bearing mice were sacrificed, and the volume of the tumours was calculated according to the generic formula: volume (mm^3^) = width^2^ × length/2. Subsequently, the tumour samples were harvested and weighed for further analysis.

### Haematoxylin–eosin (HE) and Immunohistochemistry

We fixed the samples that were collected from the mouse models in paraffin and then sectioned them to 4 mm thickness for HE staining and immunohistochemistry according to the protocols. After using the DAB (SA-HRP) TUNEL Cell Apoptosis Detection Kit, the samples were stained using terminal deoxynucleotidyl transferase-mediated dUTP-biotin nick end labelling (TUNEL) (Servicebio, China). Pressure cooking was used to extract the antigen for three minutes in a 0.01 mol L-1 citrate buffer (pH 6.0; Wobixin Inc., China). At 4 °C, the samples were incubated overnight with specific antibodies against CD3 (ab16669, 1:150 dilution; Abcam, UK), CD8 (ab217344, 1:2000 dilution; Abcam, Cambridge, UK), Ki-67 (ab16667, 1:200 dilution; Abcam, Cambridge, UK), and PD-L1 (ab213524, 1:250 dilution; Abcam, UK). The following day, we carried out immunodetection utilizing 3'-diaminobenzidine, in accordance with the instruction booklet.

### Mass cytometry

Following the death of the tumor-bearing mice, full tumor samples from the PBS and MDZ groups were collected and further processed using the Miltenyi Mouse Tumour Dissociation Kit (Miltenyi Biotec, Germany). Following are the phases in the CyTOF staining process: 194Pt staining → Fc block → surface antibody staining → overnight DNA staining (191/193Ir) → intracellular antibody staining → data collection. Two procedures make up the data analysis: FlowJo preprocessing (circle and choose individual, fully functional CD45 + immune cells), and bioinformation analysis (the X-shift algorithm performs cell subpopulation clustering, manual annotation, TSNE dimensionality reduction visual display, and statistical analysis). Most of the CyTOF staining and data processing was done by the PLTTECH Company (Plttech, China).

### Statistical analysis

The averages and standard deviations (SD) of at least three different biological replicates are used to represent all of the results. GraphPad Prism 9.0 (GraphPad software Inc., San Diego, USA) was used for the statistical analysis, and a two-tailed *P* value < 0.05 was regarded as statistically significant. The continuous variables between the two groups were compared using a two-tailed unpaired Student's t test.

## Results

### Midazolam attenuated the proliferation, invasion, and migration of HCC cell lines

**Fig. 1:**
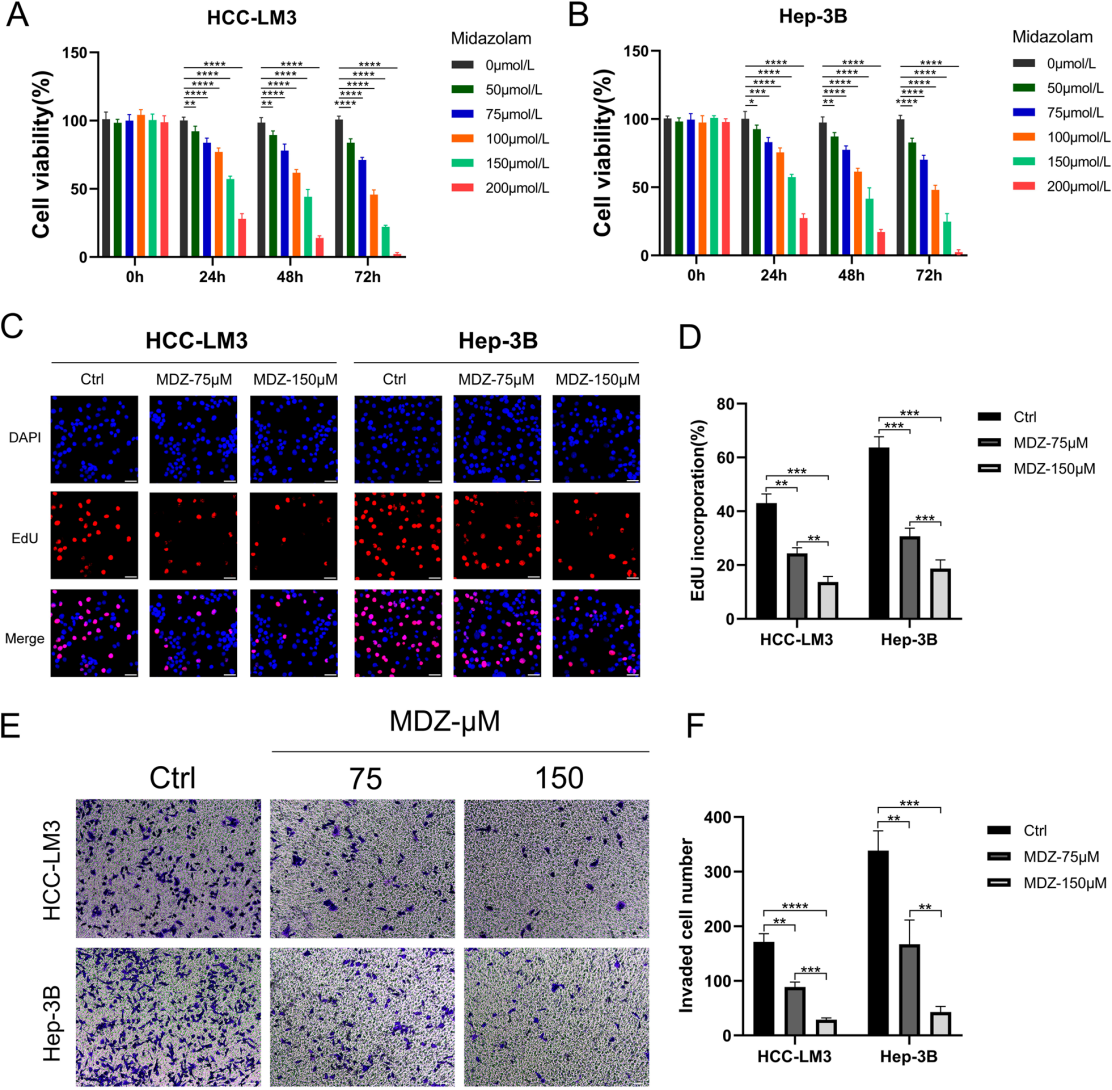
MDZ inhibited proliferation and invasion in HCC cell lines. **A**,** B** The cell viability histograms of the HCC-LM3 and Hep-3B cell lines were drawn after treatment with midazolam at increasing concentrations (0, 50, 75, 100, 150, or 200 µM) based on CCK-8 assays. **C**, **D** EdU assays were performed to assess the proliferation of HCC-LM3 and Hep-3B cells cultured with 75 and 150 µM MDZ for 24 h. Then, statistical analysis was performed on the EdU( +) proliferating cells. Cells were treated with stroke-physiological saline solution (SPSS) as the control. **E**, **F** Transwell experiments were used to assess the invasion of HCC cells incubated with MDZ. The left panel shows HCC-LM3 and Hep-3B cell invasion after MDZ treatment. The right panel shows the counts of invading cells. *P < 0.05, **P < 0.01, ***P < 0.001, ****P < 0.0001

**Fig. 2:**
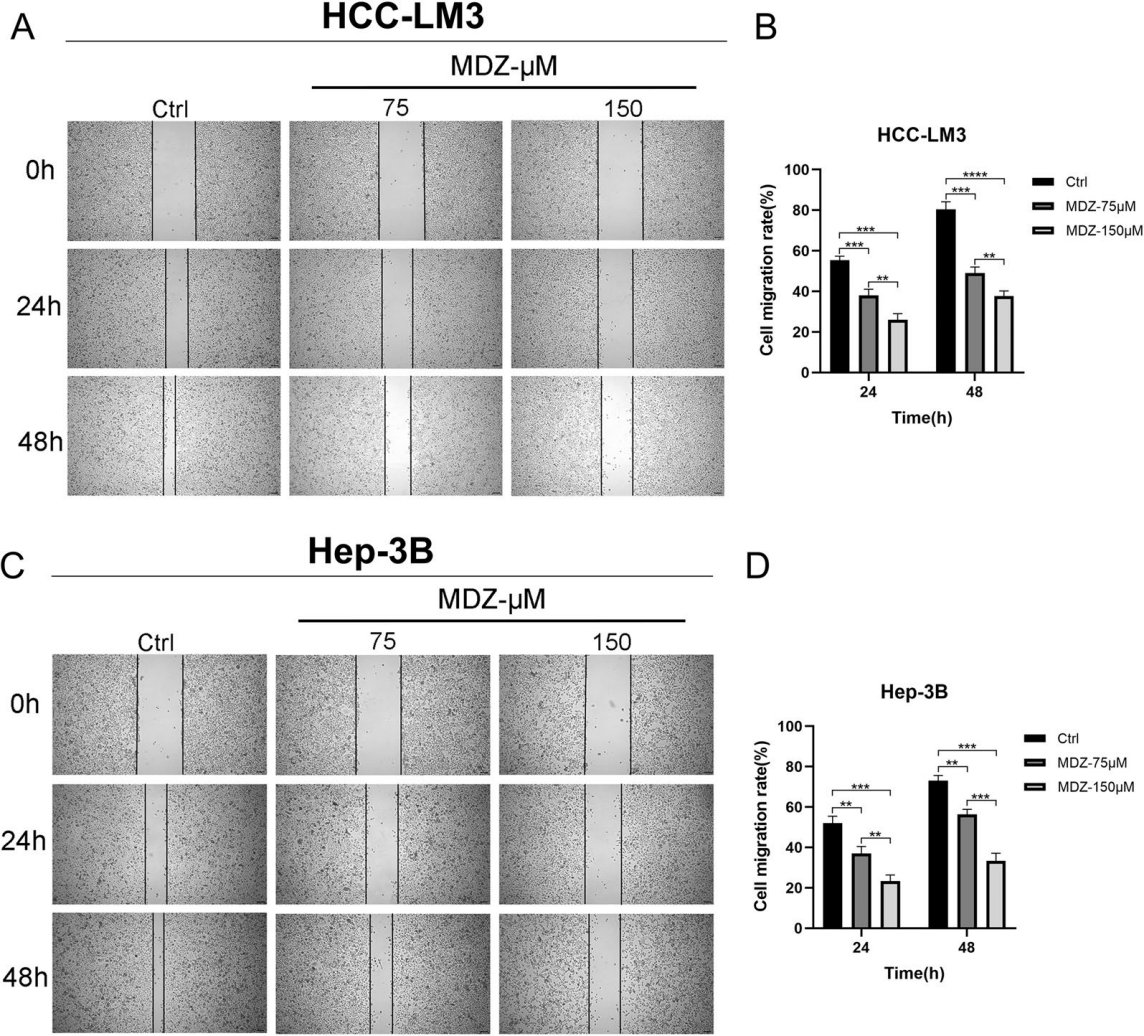
MDZ inhibited migration in HCC cell lines. **A**–**D** Wound healing assays were used to assess the migration of HCC cells treated with MDZ for 24 h and 48 h. The left panel shows HCC-LM3 and Hep-3B cell migration after MDZ treatment. The right panel shows the cell migration rate. **P < 0.01, ***P < 0.001, ****P < 0.0001

A number of in vitro tests were performed to investigate MDZ's role in HCC cells. Preliminary experiment findings (Additional file 1: Fig. S1A-B) revealed that MDZ decreased cell viability in a concentration-dependent manner at 24 h in both HCC-LM3 (IC50 = 143.2 µM) and Hep-3B (IC50 = 141.1 µM). According to the CCK8 assay, the two HCC cell lines' cell viability was dramatically reduced by MDZ in a dose-dependent manner when compared to the control. The vitality of HCC cells treated with the same quantity of MDZ decreased as the incubation duration increased compared to the controls (Fig. 1A, B). According to these experimental results, we chose doses of 75 µM and 150 µM for subsequent cell experiments. The findings of the EdU incorporation studies demonstrated that MDZ therapy dramatically reduced the number of EdU-positive cells. The findings of determining the EdU-incorporation rates of HCC cells revealed that, in comparison to the controls, MDZ considerably and dose-dependently decreased the incorporation rate (Fig. 1C, D). Additionally, we performed Transwell invasion studies. The results showed that MDZ suppressed the in vitro invasion of both HCC-LM3 and Hep-3B cells in a dose-dependent manner (Fig. 1E, F). Using a wound healing test, we also evaluated the impact of MDZ on the migration of the HCC lines. Our findings from the wound healing experiment showed that MDZ impeded the migration of both the HCC-LM3 and Hep-3B cell lines, and that the rate of wound healing fell off as MDZ concentration and incubation time increased (Fig. 2A–D). In addition, MDZ prevented HCC cells from migrating when mitomycin-C was present (Additional file 1: Fig. S2A, B). In conclusion, the results of these experiments showed that the ability of the HCC-LM3 and Hep-3B cell lines to proliferate, invade, and migrate was greatly reduced by MDZ.

### MDZ downregulated PD-L1 expression in HCC through the NF-κB signalling pathway

**Fig. 3:**
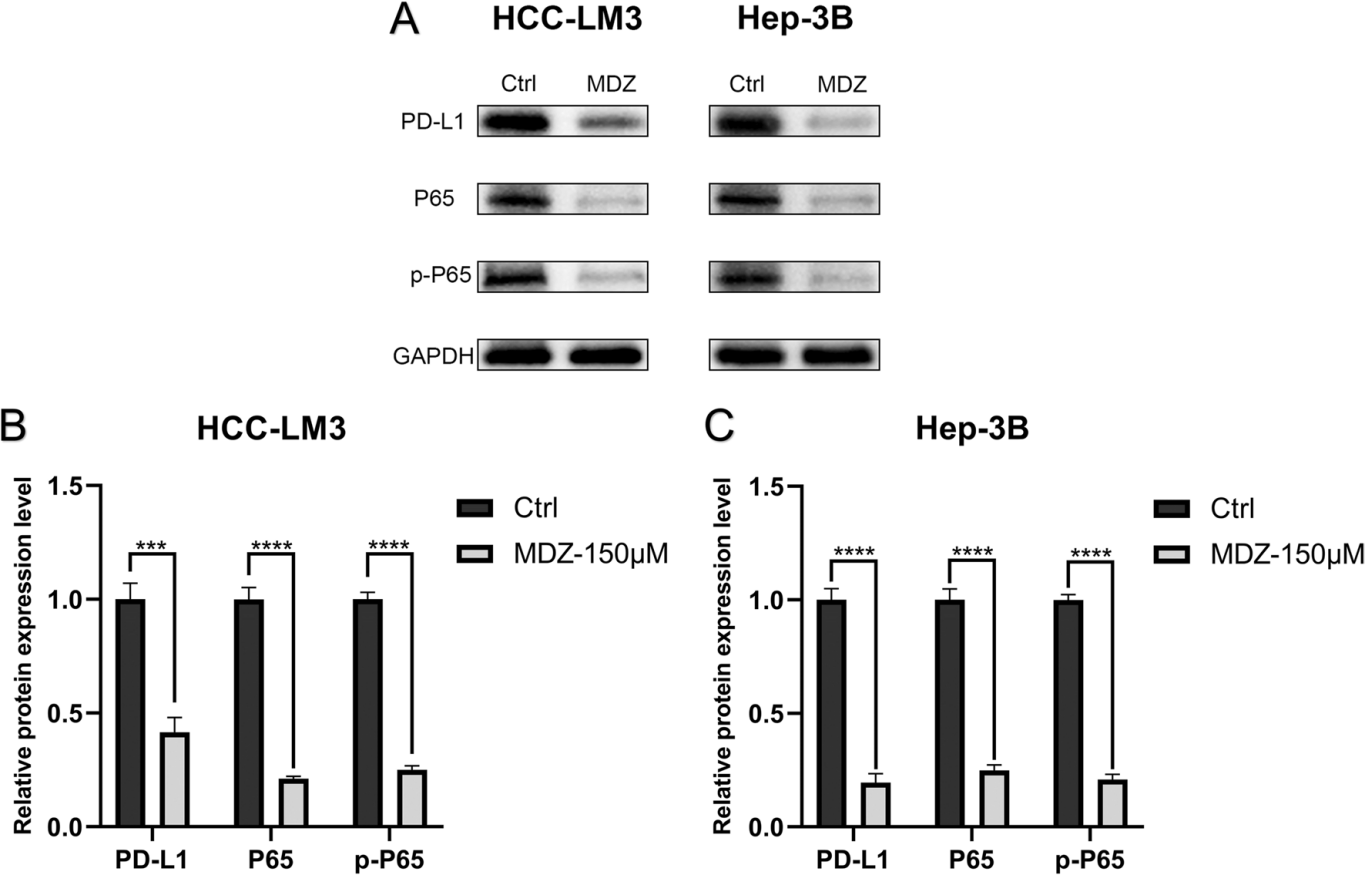
MDZ downregulated PD-L1 expression in HCC cells through the NF-κB pathway. (**A**) The protein expression levels of PD-L1, P65, and p-P65 in HCC cells after MDZ treatment. **B**, **C** The panels show the protein band grey values. ***P < 0.001, ****P < 0.0001

Our team made an effort to use PD-L1 as a first entry point to further analyze the potential mechanism in order to examine the impact of MDZ on the tumour microenvironment in HCC. To determine whether PD-L1 protein expression changed following MDZ therapy, western blotting was carried out. The results show that MDZ treatment significantly reduced the protein expression of PD-L1 in HCC-LM3 and Hep-3B cells (Fig. 3A–C).

Numerous studies [26, 27] have shown that MDZ has an impact on how NF-κB is activated. According to a prior study, during HCC immune escape, the NF-κB signaling pathway has the ability to upregulate the expression of PD-L1 [15]. To learn more about how MDZ influences the expression of PD-L1, our team came up with the hypothesis that MDZ can suppress PD-L1 expression by blocking the NF-κB signaling pathway, which would subsequently result in immunological escape in HCC. When HCC-LM3 and Hep-3B cells were treated with MDZ, the protein expression of P65 and p-P65 was considerably reduced, according to Western blotting (Fig. 3A–C). The JASPAR database predicted the binding locations and motifs of the human transcription factor P65 in the PD-L1 promoter region (Additional file 1: Fig. S3A). The human CHIP-seq data (GSE2360959) also revealed that P65 had a substantial peak upstream of PD-L1, indicating that a reachable binding site is situated there (Additional file 1: Fig. S3B). All the results suggest that MDZ downregulates PD-L1 expression in HCC through the NF-κB signalling pathway.

### MDZ inhibited tumour growth and enhanced the efficiency of PD-1 mAb immunotherapy in HCC

**Fig. 4:**
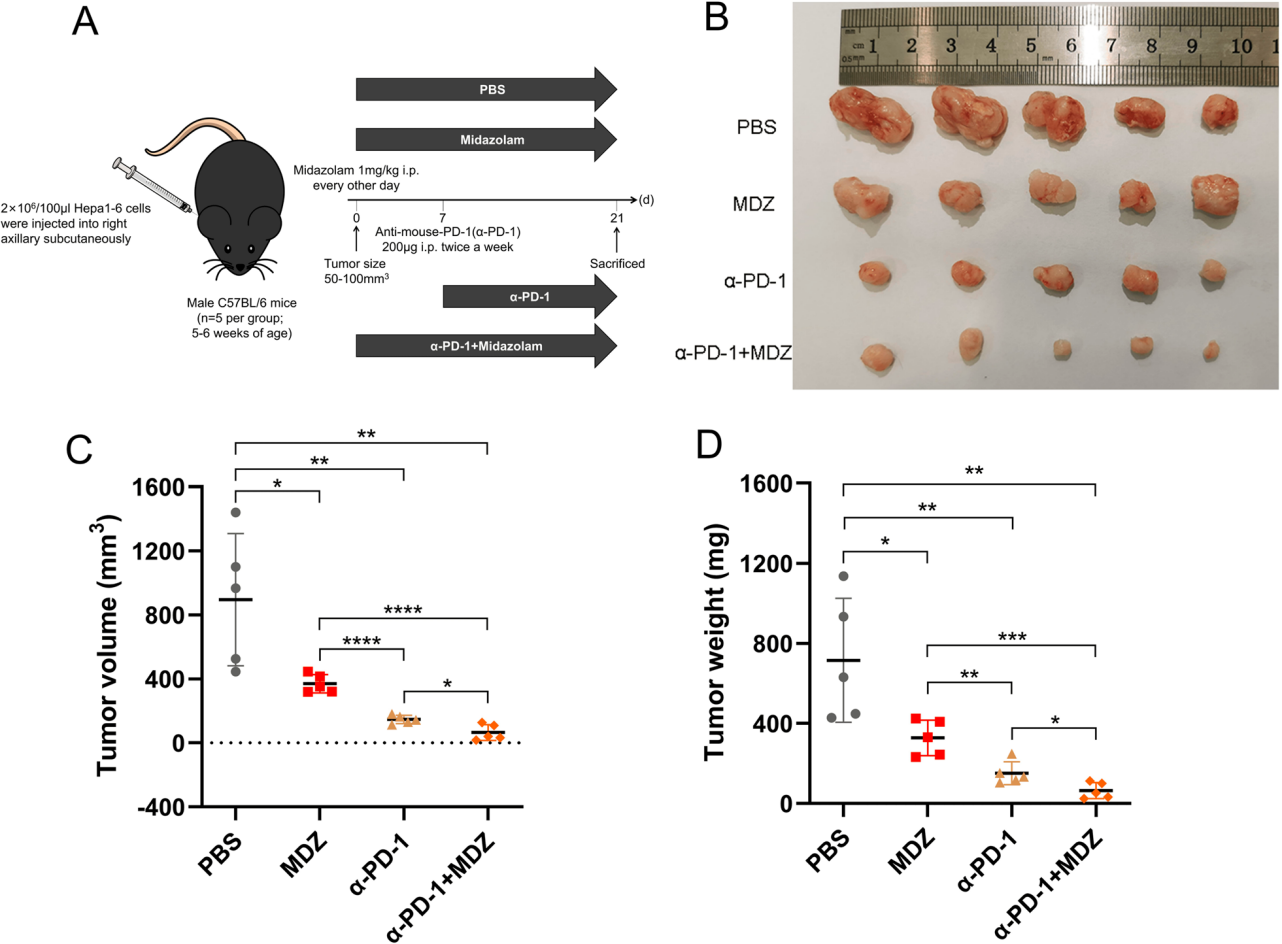
MDZ reduced tumour growth and increased the efficiency of the anti-PD-1 monoclonal antibody in a xenograft mouse model. **A** Procedures for establishing a subcutaneous tumour-bearing mouse model. PD-1 antibody was injected intraperitoneally twice a week 7 days after MDZ injection. Mice injected with PBS were the control. **B** Images of the subcutaneous tumours in the different groups (PBS, MDZ, α-PD-1, and α-PD-1 + MDZ). **C**, **D** The weights **(C)** and volumes (**D**) of the subcutaneous tumours in the indicated groups. *P < 0.05, **P < 0.01, ***P < 0.001, ****P < 0.0001

To evaluate the effect of MDZ on tumour growth and PD-1 mAb therapeutic efficacy in of HCC in vivo, we subcutaneously injected murine Hepa1-6 cells into the C57BL/6 mice in the PBS, MDZ, α-PD-1, and α-PD-1 + MDZ groups and then injected the mice with PD-1 mAb to assess the antitumor effects. More details about the experimental scheme of the tumour mouse models are provided in Fig. 4A. According to the results, compared with PBS treatment, MDZ treatment significantly decreased the tumour volumes and weights. Compared with α-PD-1 alone, combination with MDZ decreased the tumour weight and volume (Fig. 4B–D).

**Fig. 5:**
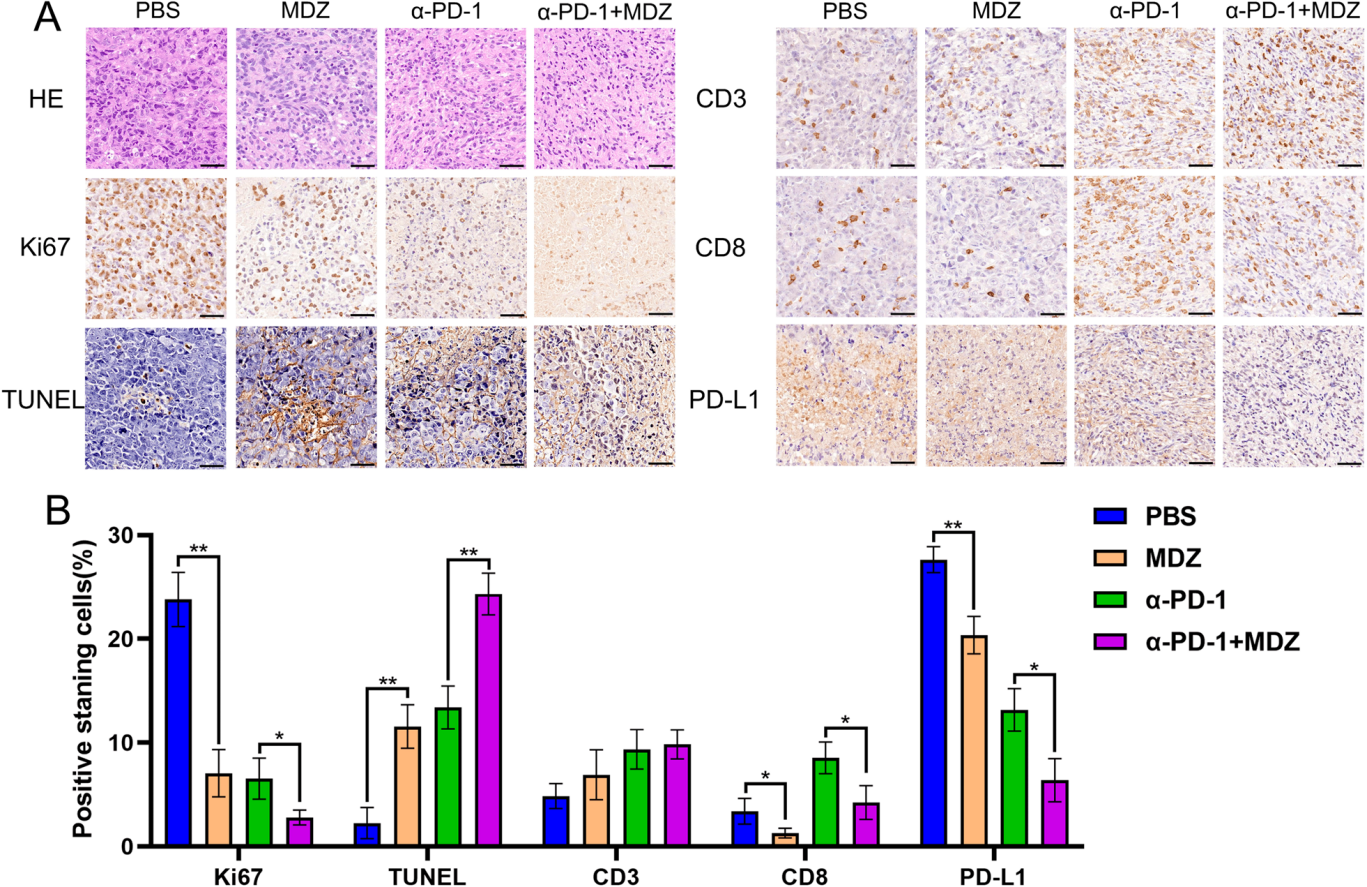
Immunohistochemistry showed that MDZ affected tumour growth and enhanced anti-PD-1 monoclonal antibody treatment efficacy in a xenograft mouse model. **A** The morphology of subcutaneous tumours in the four groups was confirmed by HE staining, as shown in the upper panel. Moreover, the upper panel indicates the results immunohistochemical staining for Ki67, TUNEL, PD-L1, CD3, and CD8 expression in the indicated groups. **B** The lower panel illustrates the statistical analysis of the levels of these indicators in the four groups. *P < 0.05, **P < 0.01

HE staining confirmed the differences in tumour tissue and morphology in the different groups. Compared with the α-PD-1 and combination groups, the cells in the PBS group were more disorganized, with enlarged and darker stained nuclei and less cytoplasmic content. Although a similar phenomenon was observed in the MDZ group, MDZ appeared to reduce the changes in the abnormal nuclear and cytoplasmic structures compared to PBS. In the α-PD-1 group, tumour cells were arranged more regularly than in the previous two groups. After coadministration of MDZ, the nuclear staining was significantly lighter, and the nuclei were smaller, with some cells having visible cytoplasm and an improved nucleocytoplasmic disproportion. Similar to the immunohistochemistry data, the MDZ group's Ki67 and PD-L1 expression was significantly lower than that of the PBS group's when compared, and the drop was noticeably aided by the use of MDZ in conjunction with PD-1 mAb (Fig. 5A, B). However, there was no discernible difference in CD3 expression following either the MDZ treatment alone or the combined treatment, while CD8 expression slightly decreased. Additionally, according to the results of the immunohistochemistry, TUNEL expression in tumor tissues was significantly higher after MDZ injections than it was in the PBS group (Fig. 5A, B), and this trend was markedly accentuated when MDZ and PD-1 mAb were used in combination. Accordingly, this work shown that, in a xenograft mice model, injection of MDZ can trigger apoptosis, inhibit tumor development, and improve the efficacy of PD-1 mAb treatment in vivo.

### Changes in the tumour immune microenvironment after MDZ treatment of HCC based on mass cytometry

**Fig. 6:**
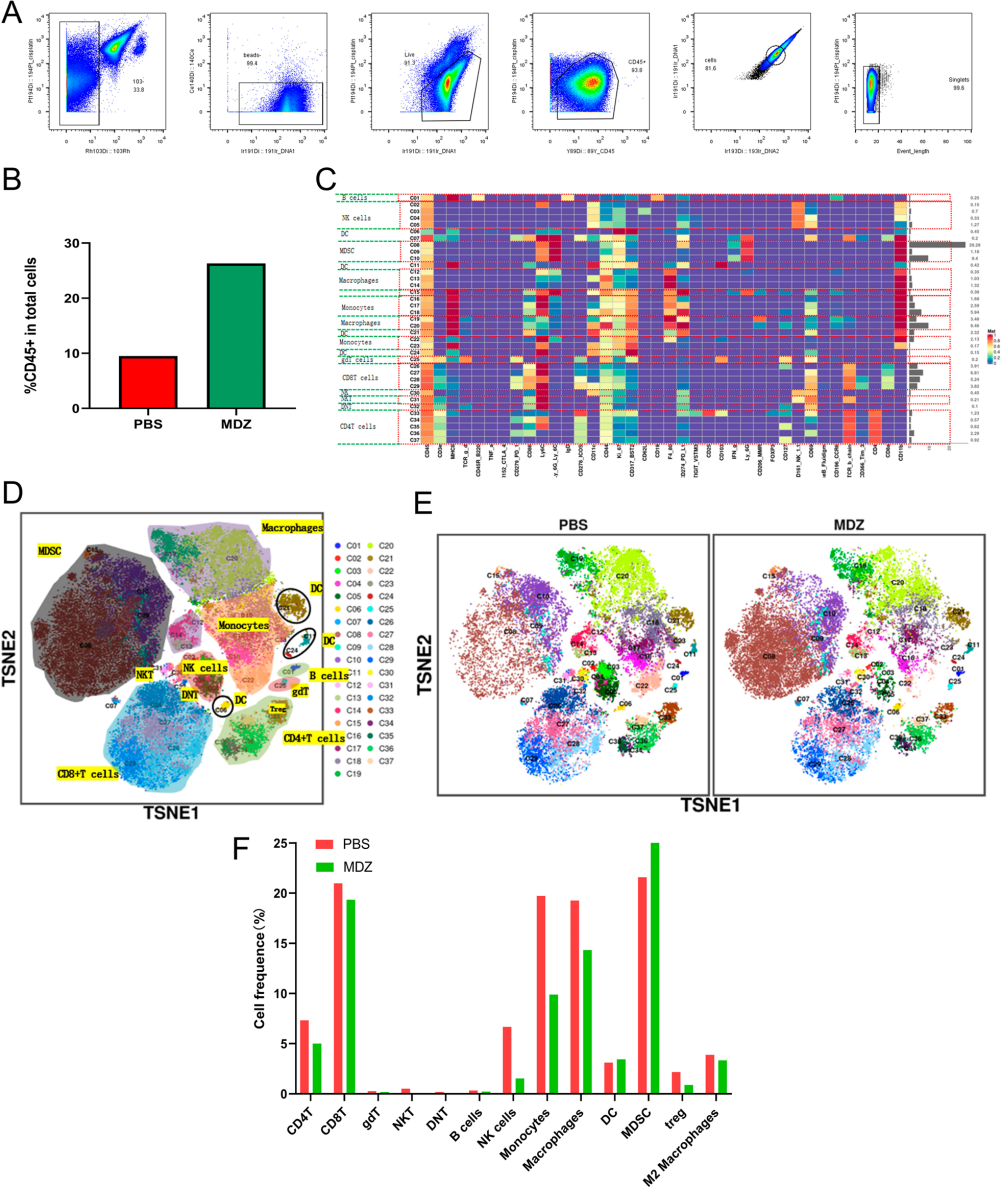
Mass cytometry was used to analyse the tumour immune microenvironment of subcutaneous HCC tumours after PBS/MDZ treatment. **A** The process of selecting CD45^+^ immune cells. **B** The proportions of CD45^+^ immune cells in different groups. **C** A total of 37 cell clusters were identified, and we defined the respective groups based on marker expression. **D** TSNE plot showing the distributions of 37 cell clusters. **E** The distribution of cell clusters in the indicated sample. **F** Histogram showing the number of the cell clusters in different groups by mass cytometry

**Fig. 7:**
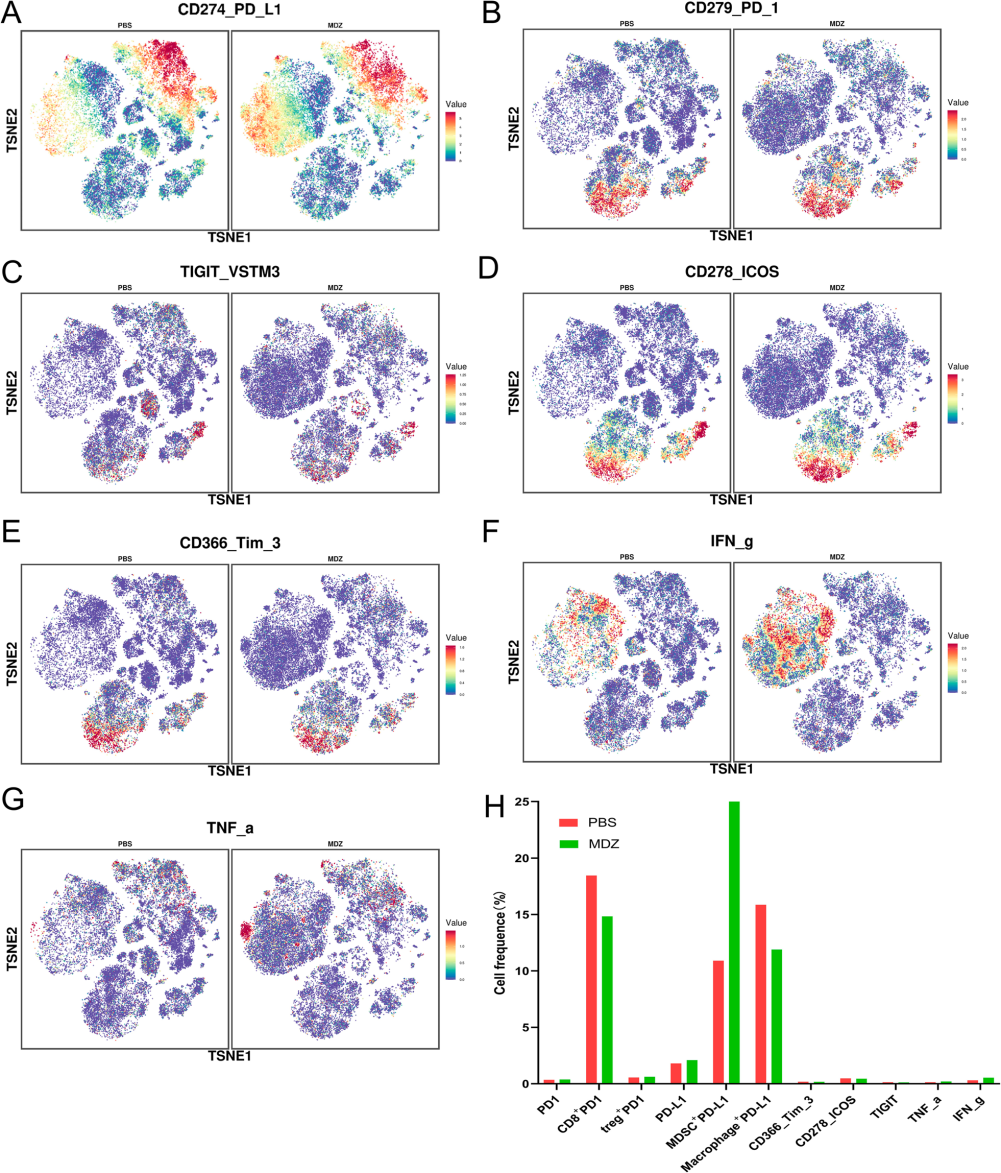
Mass cytometry revealed changes in marker expression after PBS/MDZ treatment. **A–G** TSNE plot showing the distribution of PD1, PD-L1, TIGIT, TIM3, ICOS, IFN-g and TNF-α expression in subcutaneous Hepa1-6 tumours in the PBS and MDZ groups. **H** The histogram shows the number of PD1^+^, CD8^+^PD1^+^, Treg^+^ PD1^+^, PD-L1^+^, MDSC^+^ PD-L1^+^, TIGIT, TIM3, ICOS, IFN-g^+^, and TNF-α^+^ cell clusters in the PBS and MDZ groups

Mass cytometry was used to analyze the various immune cell clusters in order to further examine the overall adjustments in the immune microenvironment of the HCC tumors in the PBS group and MDZ group. From the chosen cells in the corresponding tissues, we evaluated individual, live, and unharmed CD45^+^ immune cells (Fig. 6A). The findings revealed that the proportions of CD45^+^ immune cells in the MDZ group were higher than those in the PBS group (Fig. 6B), indicating that the immunological infiltration of the tumor was increased following MDZ treatment. CD45^+^ immune cells were annotated in subgroups and clustered across all samples. We eventually found 37 cell clusters overall after establishing the corresponding cell clusters based on certain markers of various cell types (Fig. 6C, D Additional file 1: Fig. S4). These findings demonstrate that following MDZ injection, the proportions of CD4^+^ T cells, CD8^+^ T cells, natural killer (NK) cells, monocytes, Tregs, and M2 macrophages dropped (Fig. 6E, F). Additionally, our team evaluated the overall expression of PD-L1 (CD274), PD-1 (CD279), ICOS, TIGIT, TIM3, and cytokines (IFN-g and TNF-a) in the immune microenvironment. We concluded that after the injection of MDZ, the expression of PD1 and PD-L1 in whole samples from both groups did not exhibit significant changes, possibly because of the small sample size. However, the proportions of CD8^+^ PD1^+^ cells were decreased significantly, and the proportions of MDSC^+^ PD-L1^+^ cells were increased. The levels of the exhaustion markers of CD8^+^ T cell such as ICOS, TIGIT, and TIM3, noticeably decreased, whereas the levels of cytokines (IFN-g and TNF-a) were significantly increased (Fig. 7A–H). These results suggested that MDZ injection reshaped the tumour immune microenvironment of HCC.

## Discussion

HCC is the most common tumour type of liver cancer, and its morbidity and mortality are increasing year by year in the United States and Europe [16]. ICIs are currently promising therapeutic agents for treating patients with advanced HCC and research on these agents has rapidly increased over the past decade. Unfortunately, the available evidence suggests that most HCC patients do not benefit from treatment with ICIs. In HCC patients treated with sorafenib, the overall response rate (ORR) of nivolumab treatment has been reported to be only 16–23% [17]. Therefore, finding new, potential and effective combinations of immunotherapy drugs has become one of the approaches to overcome the current lack of progress in immunotherapy.

Surprisingly, several recent studies have demonstrated that anaesthetics may have anticancer effects. MDZ belongs to the same class of benzodiazepines. Zhang et al. found that MDZ inhibited the proliferation and metastasis of hepatoma cells through the USP14/PI3K/Akt signalling pathway [18]. In addition to its sedative, hypnotic, and muscle relaxant effects, Shen et al. found that MDZ also appears to exert inhibitory effects on hepatocellular carcinoma [19]. In this study, by constructing an HCC xenograft C57BL/6 mouse model, the anti-HCC effect of MDZ in vivo was also confirmed, and this effect was significantly increased when MDZ was combined with a PD1 monoclonal antibody. However, the impact of MDZ on the tumour microenvironment and immune checkpoint blockade therapy have been less well studied. Previous studies indicate that various anaesthetics can affect the expression of PD-L1 and thereby alter the tumour microenvironment [20, 21]. Therefore, in addition to verifying the anti-HCC effect of MDZ, our study further elucidated the effect of MDZ on the HCC microenvironment and PD-L1 expression and explained the possible mechanism.

PD-L1 (B7-H1) is a ligand of PD1, and PD-L1 can be expressed in different cell types, including macrophages, dendritic cells, vascular endothelial cells, and tumour cells. Tumour cells use PD-L1 to bind to the PD1 receptor expressed by T cells, inhibiting T cell-mediated cytotoxicity in the tumour microenvironment and exerting an immunosuppressive effect [22]. Notably, in this study, MDZ was found to significantly reduce PD-L1 protein expression in HCC, as demonstrated by our experimental Western blotting results. Considering the significant effect of PD-L1 in inhibiting T-cell immune activity and immunotherapy, this study further explored the possible mechanism or pathway by which MDZ reduces PD-L1 expression. NF-κB is a group of dimeric transcription factors that play vital roles in the occurrence and development of liver inflammation and cancer. The NF-κB transcription factor family mainly includes c-Rel, RelB, p50, p52 and p65 (RelA), which usually exist as dimers [23]. Multiple studies have demonstrated that the NF-κB signalling pathway regulates PD-L1 expression and that NF-κB affects the transcription of PD-L1 by binding to the PD-L1 promoter. Mo et al. found that icaritin interacts with IKK-α to interfere with the NF-κB pathway and inhibit the expression of PD-L1 in liver cancer cells [24]. Chrysin downregulates PD-L1 expression in HCC by blocking the STAT3 and NF-κB pathways in vitro and in vivo [25]. In addition, the CHIP-seq analysis in this study revealed that in HCC, NF-κB can bind to multiple sites in the promoter region of PD-L1 and participates in regulating the expression level of PD-L1. Previous studies have examined whether MDZ can directly or indirectly affect the activation of NF-κB. For example, studies have demonstrated that MDZ can inhibit the activation of NF-κB in macrophages, thereby reducing the release of proinflammatory factors such as iNOS and COX-2 [26, 27]. According to the Western blotting results in this study, we found that the protein expression levels of P65 and p-P65 were significantly decreased in the MDZ-treated HCC cell lines. Therefore, we infer that MDZ enhances the efficacy of PD-1/PD-L1 blockade therapy by downregulating PD-L1 expression in HCC cells by inhibiting the NF-κB/p-NF-κB pathway.

The tumour microenvironment includes a complicated and changeable relational network composed of cancer cells, immune cells, stromal cells, cytokines, and extracellular matrix components, and this network is extremely important for the clinical treatment of HCC. By mass flow cytometry, we found that MDZ had a significant effect on the HCC microenvironment. Changes in the tumour microenvironment after MDZ treatment may be another critical factor that inhibits PD-L1 expression and HCC growth. In the tumour microenvironment, CD8^+^ T cells are the predominant forces that exert antitumor effects and release TNF, perforin and granzyme to kill cancer cells after activation. In addition, CD8^+^ T cells are also a significant prognostic marker of HCC [28]. By mass spectrometry measurements of common exhaustion markers of CD8^+^ T cells (TIM3, ICOS and TIGIT), we found that MDZ significantly reduced CD8^+^ T-cell exhaustion. The binding of PD-L1 and PD-1 has been demonstrated to inhibit T-cell proliferation and activation, and anti-PD-1/PD-L1 therapy can reactivate the immune response in HCC patients [29]. Therefore, the present study infers that MDZ increases the efficiency of the PD-1 mAb by inhibiting the exhaustion of CD8^+^ T cells, thereby delaying HCC progression.

Taken together, we demonstrate that MDZ shapes an immune-enhancing tumour microenvironment by restricting PD-L1 expression and reducing the depletion of CD8^+^ T cells in HCC, suggesting that MDZ is a potential antitumor drug. In the in vivo experiments in mouse models, the combination of MDZ and the PD1 monoclonal antibody achieved a better therapeutic effect than monotherapy in the treatment of HCC. MDZ-mediated inhibition of NF-κB reduced PD-L1 expression and ultimately restored the function of cytotoxic T lymphocytes (CTLs), and these effects are expected to be useful in cancer immunotherapy, providing a new direction for solving clinical problems such as PD1 resistance. However, our study also has some limitations. First, as mentioned earlier, although our study showed that MDZ prevented HCC progression and enhanced the effects of PD-1/PD-L1 blockade therapy, this still needs further clinical experimental confirmation. Second, after the injection of MDZ, the number of CD8^+^ T cells in the HCC tumour immune microenvironment decreased, and the reasons behind this need to be further explored and identified. The cytotoxicity of MDZ may affect not only tumour cells but also other immune cells, and the mechanism remains to be further studied. Furthermore, in this study, the injected doses of MDZ may be higher than those commonly used in clinical systemic administration. Long-term use of MDZ at a high dose could cause a series of side effects, such as excessive sedation, nausea and vomiting, hepatotoxicity and so on. In addition, MDZ has potential neuronal cytotoxicity. Therefore, the efficacy and safety of MDZ as an anticancer drug in clinical practice and its appropriate dose deserve further clinical study. Moreover, it is necessary to conduct more studies to evaluate the advantages and disadvantages of MDZ compared to traditional chemotherapeutic drugs in cancers, especially PD-L1-negative cancer cells.

## Conclusions

**Fig. 8:**
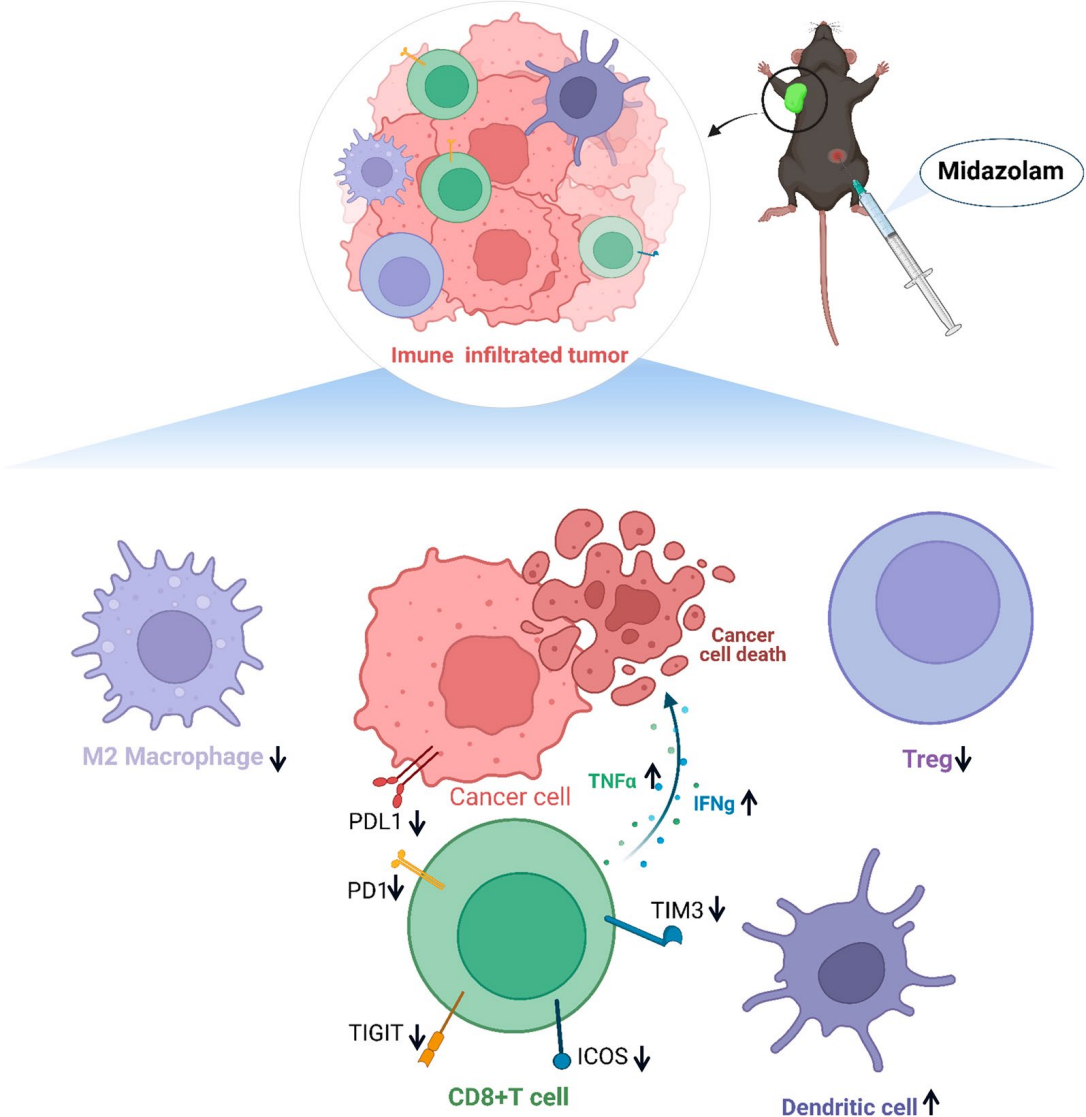
Schematic diagram of the changes in the tumour immune microenvironment of subcutaneous tumour-bearing mouse models after MDZ treatment. MDZ inhibited subcutaneous HCC tumour growth and enhanced anti-PD-1 monoclonal antibody immunity in HCC

To conclude, our study reveals that MDZ inhibits HCC tumour growth by moderating the NF-κB pathway and alleviating the exhaustion of CD8^+^ T cells. The combination of MDZ and anti-PD-1 therapy is beneficial to synergistically increase the antitumor effect of HCC therapy (Fig. 8). The present study provides a feasible idea and method for improving the therapeutic effect of anti-PD-1 therapy and provides unique insight into the association between perioperative anaesthetic management and cancer prognosis.

## Supplementary Information

**Additional file 1: Fig. S1.** The cell viability of HCC-LM3 (IC50=143.2 µM) and Hep-3B (IC50=141.1 µM) cell lines after the treatment of differential doses of MDZ at 24h. **Fig. S2.** MDZ inhibited the migration in HCC cell lines with the presence of mitomycin-C. On the basis of the original experimental methods, Mitomycin-C (1 µg/mL; GLPBIO) was present throughout the wound healing assays to avoid the interference of cell proliferation. Representative images** (A)** and quantification **(B)** of wound healing assay on HCC-LM3 and Hep-3B cell lines after the treatment of MDZ.*p<0.05, **p<0.01. **Fig. S3.** (A) The binding sites and motif of human transcription factor NF-κB in PD-L1 were predicted by the JASPAR database. (B) The human CHIP data results of the peak between NF-κB and promoter region of PD-L1(GSE2360959). **Fig. S4.** The expression of cell clustering maker genes measured by mass cytometry and presented in the form of TSNE plot.

## Data Availability

All data in this study can be obtained by contacting the corresponding author with reasonable request.
