# Supplementary material for: Midazolam exhibits antitumour and enhances the efficiency of Anti-PD-1 immunotherapy in hepatocellular carcinoma
Source: Cancer Cell Int. 2022 Oct 12;22:312. doi: 10.1186/s12935-022-02735-3 (PMC9555186; doi:10.1186/s12935-022-02735-3)

**Figure S1:The cell viability of HCC-LM3(IC50=143.2 µM) and Hep-3B (IC50=141.1 µM) cell lines after the treatment of differential doses of MDZ at 24h.**

**
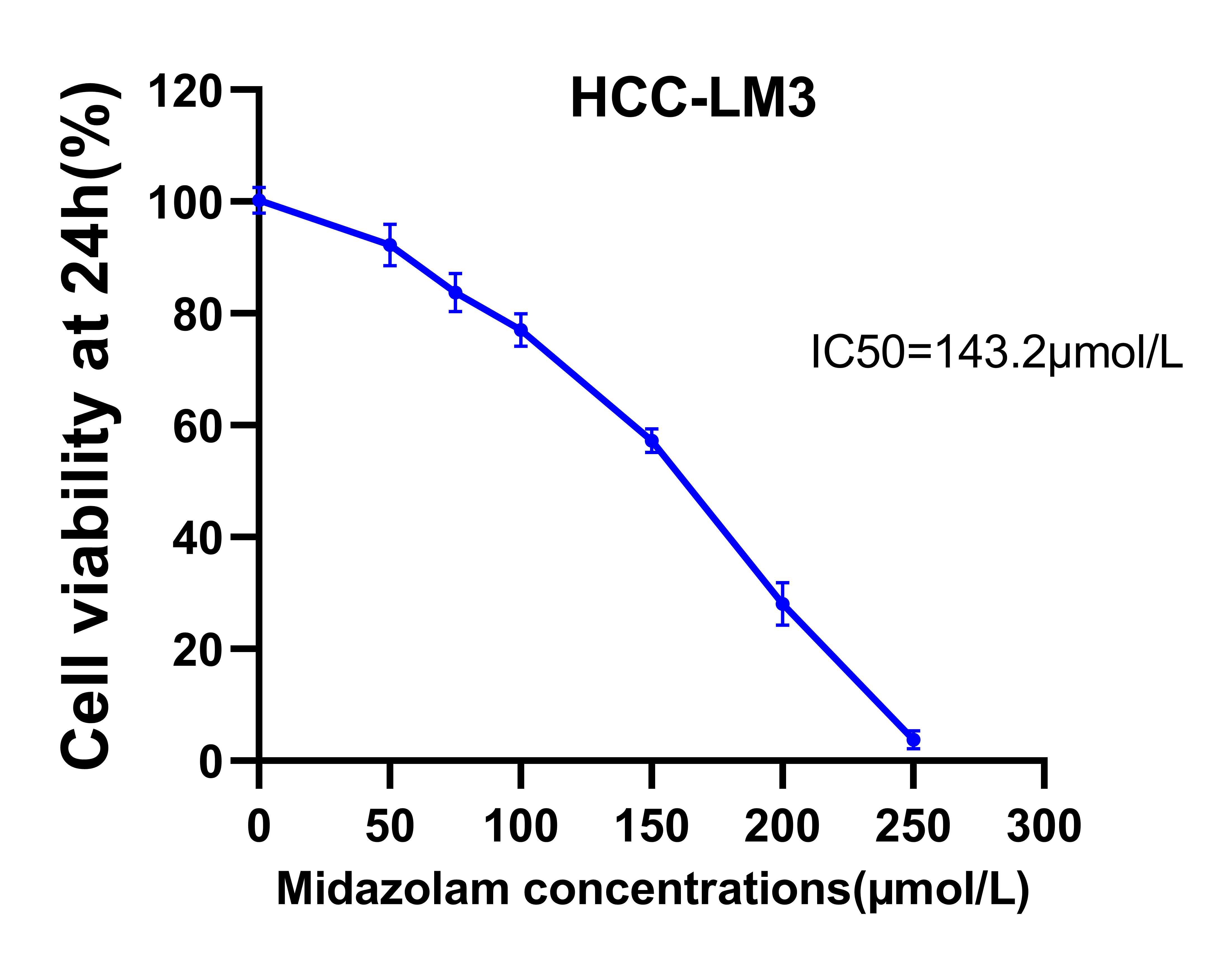

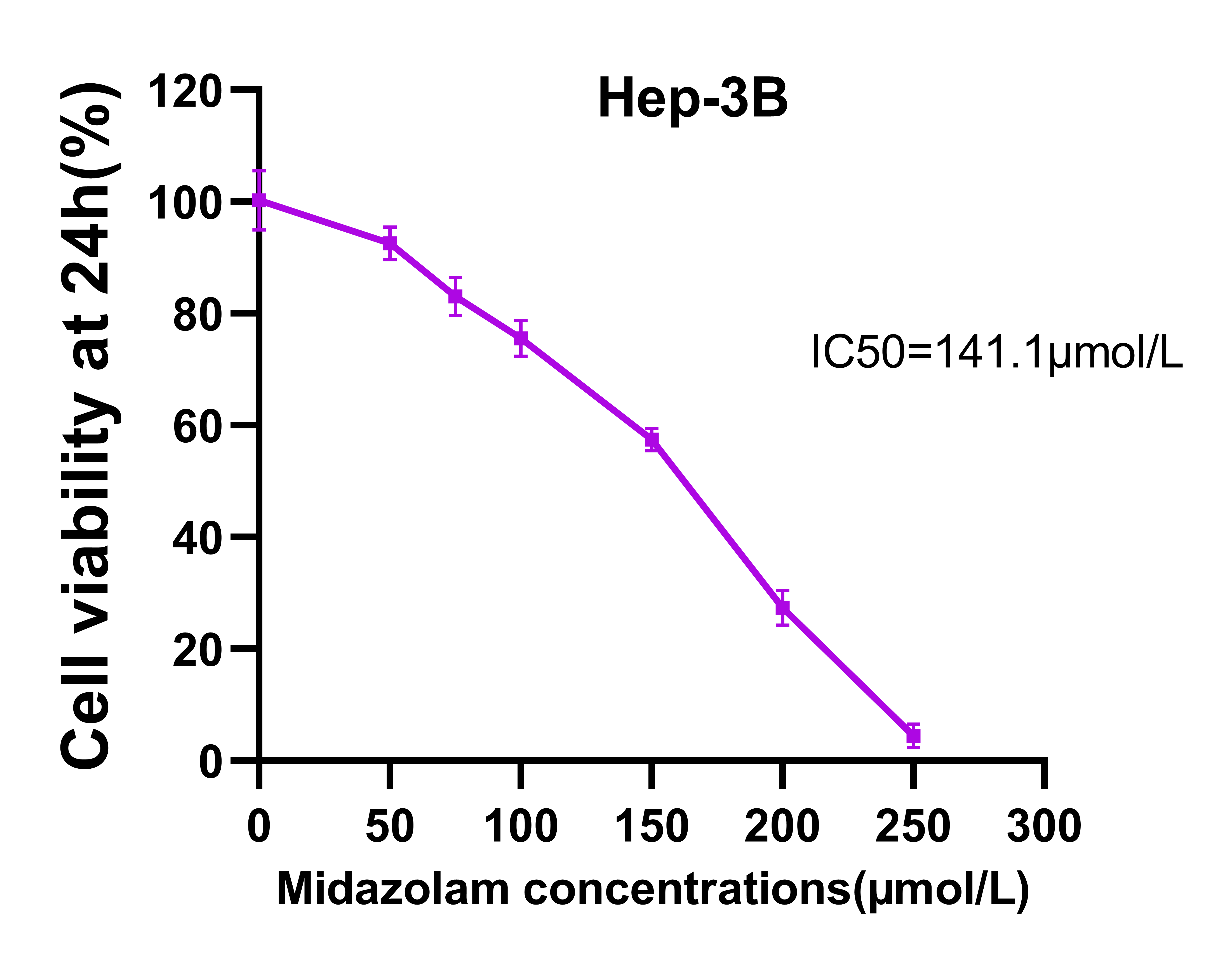
**

**Figure S2: MDZ inhibited the migration in HCC cell lines with the presence of mitomycin-C.** On the basis of the original experimental methods, Mitomycin-C (1 µg/mL; GLPBIO) was present throughout the wound healing assays to avoid the interference of cell proliferation. Representative images **(A)** and quantification **(B)** of wound healing assay on HCC-LM3 and Hep-3B cell lines after the treatment of MDZ.*p<0.05, **p<0.01.

**
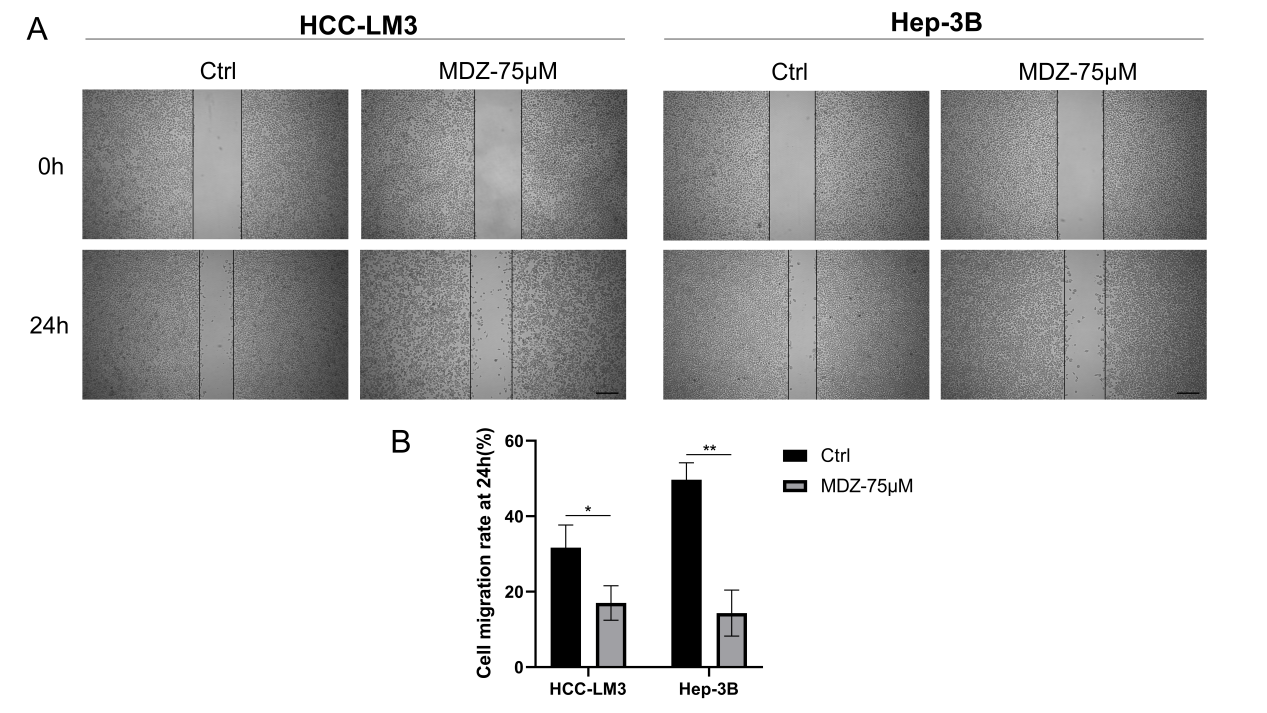
**

**Figure S3:** (**A**) The binding sites and motif of human transcription factor NF-κB in PD-L1 were predicted by the JASPAR database.(**B**) The human CHIP data results of the peak between NF-κB and promoter region of PD-L1(GSE2360959).

**A**


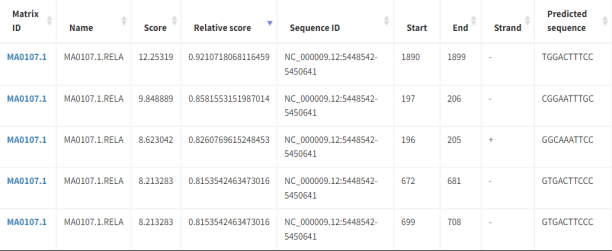

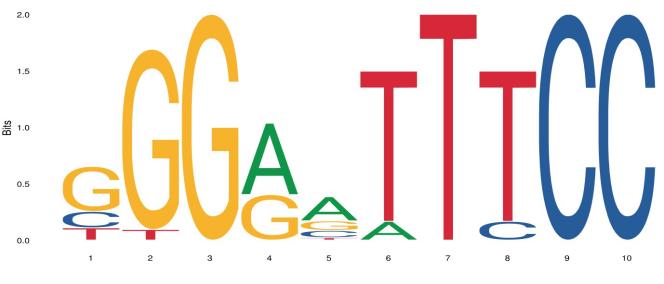


**B**


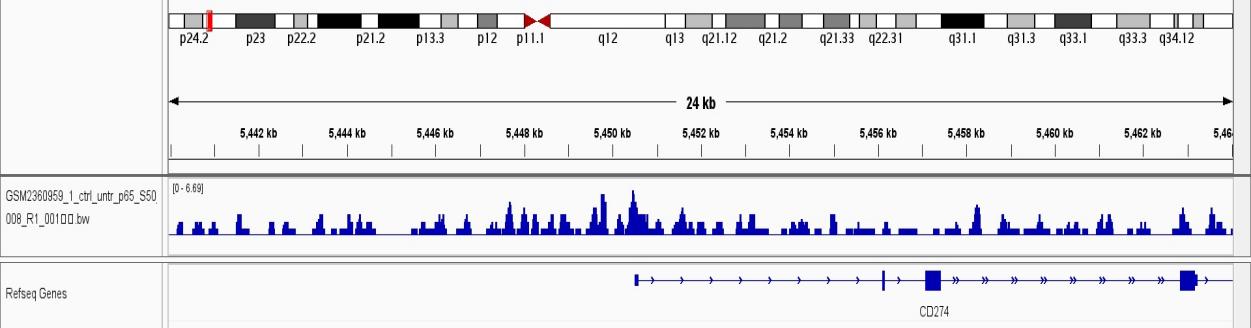


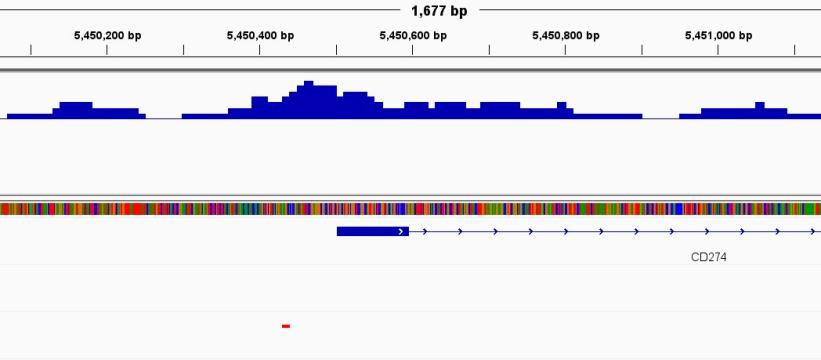


motif:TGGACTTTCC

**Figure S4: The expression of cell clustering maker genes measured by mass cytometry and presented in the form of TSNE plot.**


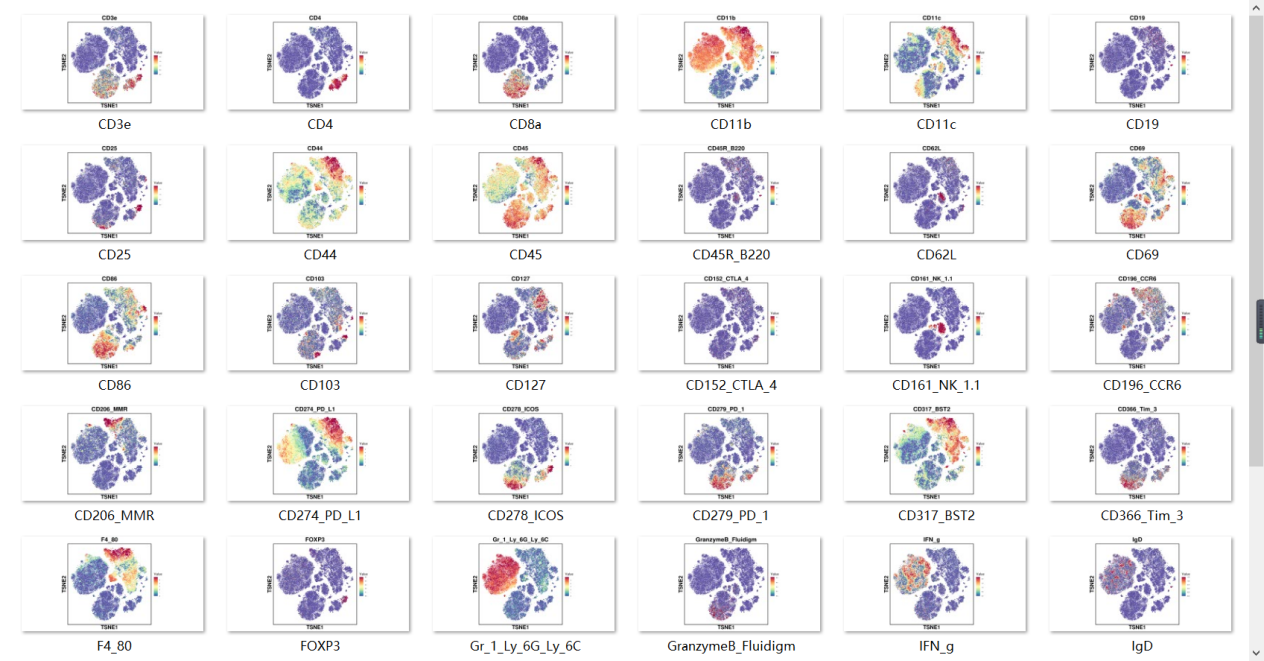

Supplement: Supplementary file 1 — Additional file 1: Fig. S1. The cell viability of HCC-LM3 (IC50=143.2 µM) and Hep-3B (IC50=141.1 µM) cell lines after the treatment of differential doses of MDZ at 24h. Fig. S2. MDZ inhibited the migration in HCC cell lines with the presence of mitomycin-C. On the basis of the original experimental methods, Mitomycin-C (1 µg/mL; GLPBIO) was present throughout the wound healing assays to avoid the interference of cell proliferation. Representative images (A) and quantification (B) of wound healing assay on HCC-LM3 and Hep-3B cell lines after the treatment of MDZ.*p<0.05, **p<0.01. Fig. S3. (A) The binding sites and motif of human transcription factor NF-κB in PD-L1 were predicted by the JASPAR database. (B) The human CHIP data results of the peak between NF-κB and promoter region of PD-L1(GSE2360959). Fig. S4. The expression of cell clustering maker genes measured by mass cytometry and presented in the form of TSNE plot. [file 12935_2022_2735_MOESM1_ESM.docx]
